# Impregnation of Scots pine and beech with tannin solutions: effect of viscosity and wood anatomy in wood infiltration

**DOI:** 10.1007/s00226-012-0524-5

**Published:** 2013-01-22

**Authors:** G. Tondi, M. F. Thevenon, B. Mies, G. Standfest, A. Petutschnigg, S. Wieland

**Affiliations:** 1 Salzburg University of Applied Sciences, Campus Kuchl, 136a Marktstraße, 5431 Kuchl, Austria; 2 Wood Preservation Laboratory, CIRAD Persyst, 73 Rue J.F. Breton, 34398 Montpellier, France

## Abstract

The impregnation process of Scots pine and beech samples with tannin solutions was investigated. The two materials involved in the process (impregnation solution and wood samples) are studied in depth. Viscosity of mimosa tannin solutions and the anatomical aspect of beech and Scots pine were analysed and correlated. The viscosity of tannin solutions presents a non-newtonian behaviour when its pH level increases, and in the case of addition of hexamine as a hardener, the crosslinking of the flavonoids turns out to be of great importance. During the impregnation of Scots pine (*Pinus sylvestris* L.) and beech (*Fagus sylvatica* L.), the liquid and solid uptakes were monitored while taking into consideration the different conditions of the impregnation process. This method allowed to identify the best conditions needed in order to get a successful preservative uptake for each wooden substrate. The penetration mechanism within the wood of both species was revealed with the aid of a microscopic analysis. Scots pine is impregnated through the tracheids in the longitudinal direction and through parenchyma rays in the radial direction, whereas in beech, the penetration occurs almost completely through longitudinal vessels.

## Introduction

Polyflavonoid or condensed tannins are natural compounds present in many plants. Within the plant kingdom, such compounds are used as preservatives offering protection against light (UV rays and free radicals) and against biological attacks (insects, fungi and bacteria) (Choi et al. 2002; Hagerman et al. 1998; De Bruyne et al. 1999).

The idea of protecting wood with wood derived natural preservatives has been known since decades (Lotz and Hollaway 1988). The main drawback of this idea has always been the high leachability of the tannin. Their extremely high solubility in water (often more than 50 % by weight) rendered these molecules unsuitable as wood preservative.

Condensed tannin is made up of oligomers constituted by the repetition of flavonoid units which are mainly linked to each other in a 4–6 or 4–8 pattern (Pizzi 1994).

This hydroxy-aromatic chemical composition has shown similar reactions to the ones found in phenols: hardeners such as formaldehyde, hexamine and glyoxal can crosslink the oligomers to produce macromolecules.

This chemical property has been the key for the development of tannins in resin formulations (Pichelin et al. 1997; Tondi et al. 2009).

The most common commercial condensed tannin is the one found in the mimosa (*Acacia mearnsii*, or *mollissima*) bark extract. This water soluble powder was already used for other applications such as wood adhesives, metal adsorbers and the production of foams (Pizzi et al. 1995; Özacar et al. 2006; Tondi and Pizzi 2009).

Based on the afore-mentioned, in situ-curing tannin–hexamine formulations were recently tested as wood preservatives for outdoor applications. The results showed that the high biological activity of these formulations, enriched with a very low amount of boron, is effective in the treatment against high-virulence tropical fungus such as *Pycnoporus sanguineus* (Thevenon et al. 2009) and termites (Tondi et al. 2012).

These very positive results have encouraged more intensive research work on the applicability of these formulations for softwood. Therefore, viscosity studies of tannin solutions, investigations concerning the impregnation process and anatomical considerations of impregnated Scots pine and beech through microscope analyses are presented in this paper.

## Materials and methods

### Materials

Wood pieces of Scots pine (*Pinus sylvestris* L.) and European beech (*Fagus sylvatica* L.) were provided from different Austrian sawmills and selected in accordance with EN113 (1997). Samples of sapwood with dimensions of 50 × 25 × 15 mm³ and oriented with the longer side along the direction of grain were after conditioning in a standard climate of 20 °C and 65 % RH to approximately 12 % equilibrium moisture content.

Commercially available Mimosa tannin extract was provided by Silva-chimica (Italy), while hexamethylenetetramine (hexamine) was provided by Lactan.

### Viscosity measurements

Tannin formulations with different concentrations up to 45 % s.c., as well as additives and pH levels were subjected to viscosity measurements. Viscosity was evaluated at room temperature (20 °C) using different spindles and observing its behaviour under different rotational speed conditions. For the test of tannin polymerisation, the solutions were immersed in boiling water (100 °C) for different amounts of time. The viscometer used was a multi-speed digital viscotester from ThermoHaake.

### Impregnation

Scots pine and European beech specimens were dried for at least 1 week at 104 °C to ensure that the samples were completely dry.

These samples were placed into a desiccator, and a vacuum of 8 mbar was applied to remove the majority of the air trapped within the wood cells. Afterwards, the desiccator was filled up with the impregnation solution and the pressure was slowly increased back to environmental pressure.

According to the different kinds of treated wood and to the viscosity of the impregnation solution, different vacuum and times of submersion were applied.

Vacuum time is the elapsed time in which the samples undergo a vacuum treatment (8 mbar), while the time of submersion is the time in which the sample is penetrated by the liquid at environmental pressure.

For some impregnations, more than one test was performed. The technique of multiple cycle impregnations is carried out by applying successive vacuum treatment to increase the penetration of the impregnating solution. The cycles used in this work were of 10 min vacuum (8 mbar) and 10 min at atmospheric pressure.

The tannin solutions used for impregnation were always corrected with NaOH 50 % to pH 9.0 and addition 6 % by weight of hexamine.

The weights of the wood specimens were gathered before and after the treatment to evaluate the amount of liquid which penetrates the sample.

The impregnated samples were kept for 12 h at 104 °C to let the tannin–hexamine resin harden in situ. The weights of the samples were determined at the end of the process to calculate the amount of trapped solids.

Impregnation rate (or retention) is the percentage of the weight ratio between the liquid uptake and the dry sample.

1 $$  {\text{I}}{\text{.R}}{\text{.}}(\% ) = (({\text{Wet}}\;{\text{weight}} - {\text{Dry}}\;{\text{weight}})/{\text{Dry}}\;{\text{weight}}) \times 100  $$

Impregnation solutions were prepared with 10, 15, 20 and 30 % w/w of mimosa tannin extract.

### Microscope analysis

Twenty per cent tannin–hexamine treated samples of Scots pine and European beech were cut into 20 × 10 × 10 mm³ pieces. The impregnated samples were softened by boiling them before the microtomy. Wood specimens were immersed in a flask of water with a few drops of glycerine (defoamer) and were boiled under reflux for different amounts of time according to the hardness of the wood species. Scots pine was ready after 3.5 h while beech samples needed longer softening time (21.5 h). The slices were finally cut under wet conditions in the three anatomical directions (transversal, radial and tangential) with a Leica SM 2000 R microtome to a thickness of 10 μm. The three sections were observed with a Nikon eclipse E200 optical microscope.

## Results and discussions

The impregnation by tannin solutions was studied for Scots pine and beech samples. A complete overview of the penetration mechanism can be obtained by focusing on both elements: the impregnation solution and the wooden substrate.

### Study of the tannin solution

The most interesting feature for the impregnation solution is viscosity. Mimosa extract water solutions, indeed, have a viscosity that depends on solid content, pH and temperature.

When maintaining a constant temperature of 20 °C, the viscosity tendency was evaluated by changing the solid content and the pH of the tannin solutions.

**Fig. 1:**
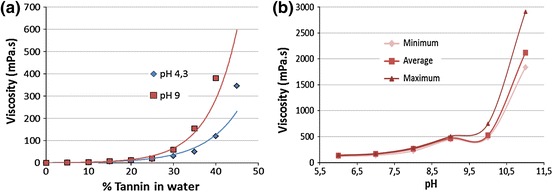
**a** Viscosity as a function of tannin solid content for pH 4.3 and 9.0 **b** viscosity as a function of pH for highly concentrated tannin solutions (45 % s.c.) (colour figure online)

In Fig. 1a, the trend of viscosity for two different pH is reported: 4.3 that is the natural pH of a mimosa tannin solution and 9.0 that is the value applied in the impregnation process in order to have a good compromise between low viscosity and high reactivity. The graphic shows that viscosity is exponentially proportional to the amount of dissolved solid, and when pH is buffered at 9, the slope of increasing viscosity occurs for less concentrated solutions.

Considering highly concentrated solutions (45 % tannin), the viscosity tendency was monitored with stepwise increasing pH (Fig. 1b). It can be observed that the viscosity increases exponentially with the pH, and when the pH reaches values around 9, the measurement of the viscosity becomes dependent on the stirring rate of the viscometer spindles. The minimum as well as the average and the maximum value of viscosity evaluated with different stirring rates are reported in the graphic. This lack of uniformity in viscosity measurements is typical of non-newtonian fluids, and in particular, such kind of materials can be classified as pseudoplastic.

From the chemical point of view, new intermolecular bonding takes place in alkaline environment and the oligomeric solution increases its molecular weight. In fact, solutions with high molecular weight often have non-newtonian behaviour.

The strongly alkaline environment, indeed, activates the hydroxyl groups of the flavonoids to autocondensation (Pizzi 1981).

**Fig. 2:**
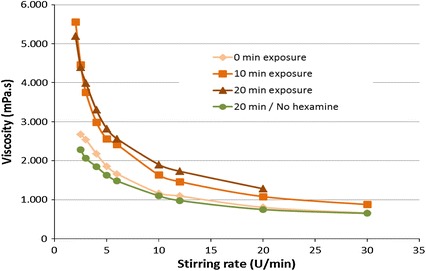
Viscosity as a function of stirring rate for a 45 % tannin solution with 6 % hexamine at pH 11 for different exposure times at 100 °C boiling water (colour figure online)

The polymerisation of flavonoids was then evaluated for different exposures to boiling water (100 °C) with and without hardener. Considering the solutions with high viscosities (45 % s.c. and pH = 11), some thermal-curing tests were done to monitor the crosslinking behaviour of the formulations. The viscosity was measured for different exposure of the sample at 100 °C (Fig. 2). It can be generally observed that the viscosity decreases when the stirring speed increases (pseudoplastic non-newtonian behaviour).

The first parameter to be considered is the effect of the hardener. The viscosity curve of the tannin solution without hardener is, indeed, very similar to the one where no heat is supplied. This means that activation time does not affect the system if no hardener is added.

Conversely, the viscosity behaviour changes when hexamine is added to the formulation. Hexamine, indeed, crosslinks with flavonoids and promotes the polymerisation with consequent increasing of molecular weight. This polymerisation is catalysed by heat, hence, the viscosity of tannin–hexamine solutions increases proportionally with the time of exposure.

The non-newtonian behaviour is evident for highly viscous solutions but it cannot be easily observed when the tannin solutions are diluted.

For this reason, it is still possible to consider the viscosity of tannin solutions as constant during the impregnation of the wood samples and the viscosity of a 20 % tannin solution is 8–10 mPa s at room temperature.

However, when the time of submersion becomes significant (24 h or more), some crosslinking effects have to be considered and the penetration in the wooden samples should be affected.

### Study of the impregnation in wood

Once the aspect of the liquid had been clarified, the procedure to impregnate wood was investigated. Ten and 20 % solutions of tannins were used to impregnate Scots pine and beech samples modifying vacuum and time of submersion to find out the optimal conditions for the impregnation.

**Fig. 3:**
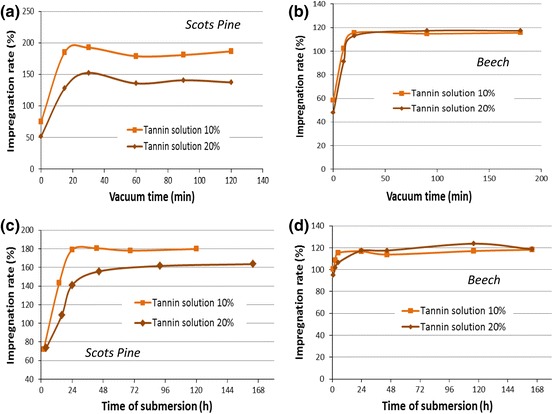
Impregnation rates of wood samples: as a function of **a** vacuum applied for 10 and 20 % solutions for Scots pine **b** vacuum applied for 10 and 20 % solutions for beech **c** time of submersion for 10 and 20 % solutions for Scots pine **d** time of submersion for 10 and 20 % solutions for beech (colour figure online)

In Fig. 3, the impregnation rate of Scots pine and beech with fixed condition of vacuum time and time of submersion can be seen.

Vacuum time was evaluated by keeping the time of submersion fixed at 24 h. When comparing Fig. 3a, b, it can be observed that after application of 20 min of vacuum, the penetration can be considered complete. Only in the case of the solution with 20 % tannin for pine, the penetration is incomplete, but increase in vacuum time does not improve the impregnation rate.

In Fig. 3c, d, the effect of the time of submersion is reported when 3 h of vacuum time are applied. It can be observed that the impregnation goes slowly for the Scots pine samples. Within 24 h, complete penetration can be obtained only for diluted tannin solutions (10 %). In the case of solutions with 20 %, the maximum uptake can be reached after more than 2 days of impregnation. For beech, a few hours are already enough to obtain significant impregnation rates, but the process can only be considered complete when the time of submersion reaches 24 h.

The conditions for successful impregnation of Scots pine can be further developed by means of a complementary study where less pressure and submersion are applied.

Scots pine specimens were impregnated with 10 % tannin solutions, and the parameters for impregnation (vacuum time, time of submersion and amount of cycles) were investigated by applying milder conditions in order to optimise the process.

In Table 1, various impregnations and their relative retention are shown.

**Table 1:** Impregnation rates for Scots pine samples when impregnated with 10 % tannin solution at mild conditions

Vacuum treatment			Impregnation rate (%)	Standard deviation
Vacuum time (min)	Time of submersion (min)	No. of cycles		
10	60	1	**117.5**	18.6
20	60	1	**116.3**	11.1
30	60	1	**129.0**	12.5
10	10	1	**61.6**	5.1
10	30	1	**100.3**	21.3
10	60	1	**117.5**	18.6
10	120	1	**129.9**	5.1
10	240	1	**140.7**	7.3
10	1440	1	**166.9**	7.9
10	10	1	**61.6**	5.1
10	10	2	**64.8**	4.1
10	10	3	**65.4**	12.9

The effect of vacuum evaluated by applying 1 h of submersion shows a clear increase, even if the data are affected by a high standard deviation.

**Fig. 4:**
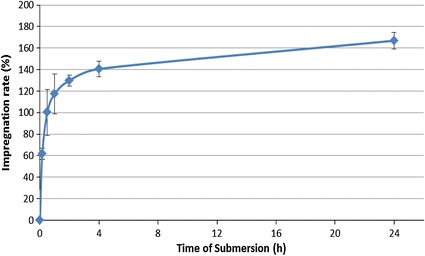
Kinetic of Scots pine impregnation with a 10 % tannin formulation (colour figure online)

The effect of the time of submersion is even more evident when only 10 min of vacuum are applied. This series of impregnations clarifies the kinetics of the impregnation process, reported in Fig. 4.

This diagram shows the importance of the first phase of the impregnation. The gradient of the initial stage of the curve is very steep and indicates that the greater uptake takes place in the first 2 hours of submersion. However, the impregnation requires a longer period of time until it can be considered complete.

In the last three rows of Table 1, the effect of multiple cycles is shown. Even if the impregnation rate increases slightly, the effect of the cycles remains limited. If considering that the samples that undergo three cycles were dipped in the solution for 30 min, the impregnation rate is much lower than the one that undergoes 30 min of submersion after a single vacuum cycle. In the first phase of the penetration process, the cycles are not required.

**Fig. 5:**
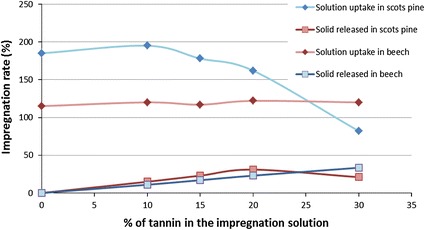
Impregnation rate of liquid penetration and solid released for different concentrations of tannin in the impregnation solution in Scots pine and beech (colour figure online)

In Fig. 5, solution uptake and related solid released for 0, 10, 15, 20 and 30 % tannin solutions are reported. These values were registered when the most effective impregnation conditions had been applied (high vacuum time and time of submersion).

The effect of viscosity of the solution significantly affects the penetration in Scots pine, while for beech, the amount of tannin does not influence the impregnation rate (at least up to 30 % s.c.).

In terms of released solid, it can be seen that the solution of 20 % s.c. represents a threshold for pine samples of 50 × 25 × 15 mm³.

The solid released is an important issue. Of course, highly concentrated solutions release high amount of solid in the wooden structure, but their higher viscosities deny the possibility of deep penetration.

Therefore, different formulations have to be chosen according to the final application of the specimens. Highly viscous concentrated solutions have to be preferred for surface treatment, while low concentrated solutions are more useful for massive-long term treatment as well as for outdoor uses.

The penetration of tannin solutions with the same concentration and at the same impregnation conditions in Scots pine and beech is different. This means that the wood anatomy of these species plays a key role in the explanation of the impregnation process.

**Fig. 6:**
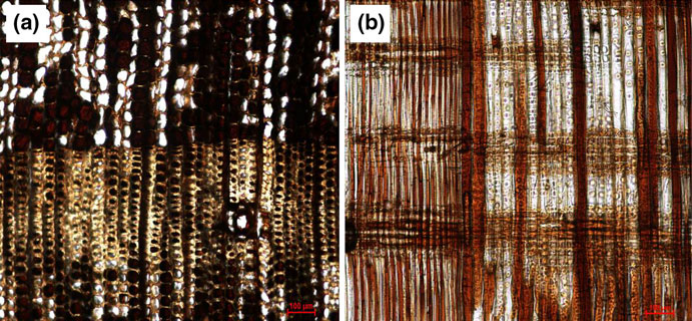
×10 Microscope images of impregnated Scots pine: **a** transversal section **b** radial section (colour figure online)

Microscope pictures of transversal and radial sections of a fully impregnated Scots pine sample are reported in Fig. 6a, (transversal) b (radial).

Some considerations can be observed:

- Tracheids are penetrated. Full impregnation can be observed for the most part of the latewood, while partial impregnation affects the earlywood.
- Tracheids are impregnated mostly when they are in an adjacent position to parenchyma rays.
- The impregnation affects certainly the parenchyma rays. Almost all of them are entirely filled by the impregnation solution.
- Resin canals are never penetrated by tannin solutions.

The longitudinal penetration through tracheids is easier for latewood because its bordered pits are rarely closed (Bamber and Burley 1983; Liese and Bauch 1967) while the bordered pits of earlywood are often closed and only wet conditioning of the samples or strong vacuum-pressure cycles would allow passing of the liquid through these pits. When more viscous tannin solutions are applied, there is a decrease in the capillary effect (especially for latewood tracheids) which explains the lower impregnation rate.

The penetration of the parenchyma rays is not dependent on the viscosity because their average thickness of around 150–200 μm allows the passage of tannin solution. Anyway, the majority of the penetration is longitudinal and if this penetration is weak the impregnation rate is low.

Some studies of lateral penetration of Scots pine and beech have recently been done by Scholz et al. (2010) where the main role of parenchyma rays in radial penetration is well described.

**Fig. 7:**
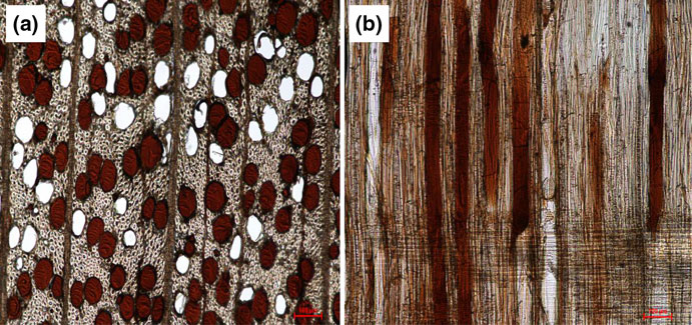
×10 Microscope images of impregnated beech **a** transversal section **b** radial section (colour figure online)

The microscopic investigation of beech is depicted in Fig. 7a, b.

In the case of beech, the impregnation takes place almost exclusively across the longitudinal direction through the large and easily accessible vessels. In the radial cut, it is possible to underline that not all the vessels are entirely filled due to the dimensions of the cells. In the middle of the specimen, only the larger vessels are impregnated because the smaller ones can be obstructed by the bigger tannin oligomers. However, the high viscosity of the tannin solution (up to 30 % s.c.) does not modify significantly the penetration in beech. The porous structure of European beech ensures an easy and deep impregnation by tannin solutions.

Finally, the pictures of impregnated Scots pine and beech show that the process does not nick the cell walls (Figs. 6, 7).

The tannin solution penetrates into the cell and is stored in the lumen. Indeed, the molecules of these oligomers are too large to establish chemical bonding with the hemicelluloses of the cell walls. Possible interactions can be assumed with the interface lignin molecules because several studies of polymerisation between tannin and lignin have been performed (Lei et al. 2008; Mansouri et al. 2010) but no significant scientific evidence has been published to date.

## Conclusion

The viscosity of tannin was studied in detail, and its importance in wood impregnation is demonstrated. It was seen that in the case of waterborne tannin solutions, the penetration is quite easy for beech while the treatment of Scots pine needs more attention.

Scots pine preservation with tannin-based formulations occurs successfully when 10 % low viscosity solutions are applied, but when formulations with higher concentration are applied, the complete impregnation rate will not be achieved.

Kinetic studies of the penetration of Scots pine have shown that the maximum uptake occurs in the first 2 h of the process.

Microscopic analysis showed that penetration in Scots pine occurs longitudinally through tracheids with open bordered pits and across radial direction through parenchyma rays. Beech is almost exclusively penetrated in the longitudinal direction through large and easy accessible vessels.

High-potential tannin-based formulations are suitable for a new generation of environment-friendly wood preservatives.
